# MiR-92b and miR-9/9* Are Specifically Expressed in Brain Primary Tumors and Can Be Used to Differentiate Primary from Metastatic Brain Tumors

**DOI:** 10.1111/j.1750-3639.2008.00184.x

**Published:** 2009-07

**Authors:** Dvora Nass, Shai Rosenwald, Eti Meiri, Shlomit Gilad, Hilla Tabibian-Keissar, Anat Schlosberg, Hagit Kuker, Netta Sion-Vardy, Ana Tobar, Oleg Kharenko, Einat Sitbon, Gila Lithwick Yanai, Eran Elyakim, Hila Cholakh, Hadas Gibori, Yael Spector, Zvi Bentwich, Iris Barshack, Nitzan Rosenfeld

**Affiliations:** 1 Departmentof Pathology, Sheba Medical CenterTel-Hashomer, Israel; 2 Sackler School of Medicine, Tel-Aviv UniversityTel-Aviv, Israel; 3 Rosetta Genomics Ltd.Rehovot, Israel; 4 Soroka University Medical CenterBeer-Sheva, Israel; 5 Department of Pathology, Beilinson hospital, Rabin Medical CenterPetah-Tikva, Israel; 6 Pathology Institute, Sourasky Medical CenterTel Aviv, Israel

**Keywords:** MicroRNA expression, Molecular diagnostics, Tumor classification

## Abstract

A recurring challenge for brain pathologists is to diagnose whether a brain malignancy is a primary tumor or a metastasis from some other tissue. The accurate diagnosis of brain malignancies is essential for selection of proper treatment. MicroRNAs are a class of small non-coding RNA species that regulate gene expression; many exhibit tissue-specific expression and are misregulated in cancer. Using microRNA expression profiling, we found that hsa-miR-92b and hsa-miR-9/hsa-miR-9* are over-expressed, specifically in brain primary tumors, as compared to primary tumors from other tissues and their metastases to the brain. By considering the expression of only these two microRNAs, it is possible to distinguish between primary and metastatic brain tumors with very high accuracy. These microRNAs thus represent excellent biomarkers for brain primary tumors. Previous reports have found that hsa-miR-92b and hsa-miR-9/hsa-miR-9* are expressed more strongly in developing neurons and brain than in adult brain. Thus, their specific over-expression in brain primary tumors supports a functional role for these microRNAs or a link between neuronal stem cells and brain tumorigenesis.

## INTRODUCTION

A current effort of cancer research is to discover biomarkers that can improve cancer diagnosis in a clinical setting. Specifically, the pathological characterization of brain malignancies remains a diagnostic challenge. Despite the advent of various high throughput genomic-level technologies, which allow multiple DNA sequences, mRNAs or proteins to be evaluated simultaneously and systematically, these have had little impact on clinical procedures. Recently, however, there has been a paradigm shift in our understanding of genome expression, with the realization that there exists a class of small non-coding RNA species, known as microRNAs (miRNAs or miRs), which have critical functions in many biological processes (27). Although the total number of microRNAs remains controversial (2, 3, 18) and the roles of specific microRNAs are only beginning to be defined, high throughput microRNA expression analyses indicate that these species represent promising candidates for clinical tumor cell markers (6).

In general, microRNAs are regulated and transcribed similarly to protein coding genes. Subsequent microRNA biogenesis involves discrete processing and transport steps, whereby the active moiety of 20–22 nucleotides is excised from a longer RNA precursor that exhibits specific hairpin structure. Finally, these 20–22 nucleotides are incorporated into a composite machinery, termed the RNA-induced silencing complex, which promotes partial duplex formation between the short RNA and the 3′ untranslated regions of targeted transcripts, resulting typically in mammals in translational silencing (27). Systematic, high throughput microRNA expression analyses of many diverse tumors indicate that tumors display microRNA expression profiles that are significantly different from those of normal tissue, and moreover, these microRNA profiles are extremely informative with respect to developmental lineage and differentiation state of the tumor (6). Studies have suggested that, unlike with mRNA expression, a modest number of ∼200 microRNAs might be sufficient to classify human cancers (15). According to emerging studies that reveal an unexpected target specificity of microRNAs (11), we set out to investigate whether a much smaller number of microRNAs may serve to identify tumors and to define clinically important tumor characteristics. In a recent study (23), we used microRNA microarray data and developed general classification algorithms for a range of tissues. In that study, we also identified microRNAs that can identify brain tumors from a subset of non-epithelial tumors.

Differentiation between primary and metastatic tumors in the brain is often encountered in pathological practice, as metastatic tumors to the brain are quite frequent. The most common tumors to metastasize to the brain originate in the lung(10), breast (30) and skin (melanomas) (5, 7, 22); their respective contributions to all central nervous system (CNS) metastases are 30%, 20% and 10%. Although rare, choriocarcinoma disseminates to the brain with a particularly high frequency (26). In autopsy studies, 24% of cancer patients exhibited metastatic tumors in the CNS (19). Indeed, surgical pathologists are regularly presented with specimens from patients with a history of systemic neoplasia but with findings that suggest a primary intracranial tumor (19).

Here, we directly compare brain tumors to a wide range of epithelial tumors and metastases to the brain. Using microarray data, we found that elevated expression of just two microRNAs, hsa-miR-92b and hsa-miR-9*, is sufficient to distinguish brain primary tumors from tumors derived from non-brain tissues, and most significantly for diagnostic purposes, from metastases located in the brain. We translated this assay to a qRT-PCR platform, using additional samples as a training set to develop a classifier. Validating on an independent set of test samples, we found that the simple combination of hsa-miR-92b and hsa-miR-9 (or hsa-miR-9*) can identify brain metastases from brain primary tumors with sensitivity of 88% and specificity of 100%. Thus, economical and relatively easy evaluation of hsa-miR-92b and hsa-miR-9/9* expression, which can be performed robustly using either fresh frozen or fixed materials in the clinical setting (33), reveals whether neoplastic tissue excised from the brain is brain-derived or represents a metastasis from another tissue. Hsa-miR-92b and hsa-miR-9/9* were identified previously to be over-expressed in developing brain and neuronal stem cells compared to adult brain, and thus have been implicated as players in human nervous system development (12, 13, 32, 34). Taken together, the expression data concerning hsa-miR-92b and hsa-miR-9/9* suggest a connection between deregulation of microRNAs, pluripotency and tumorigenesis (4, 31).

## MATERIALS AND METHODS

### Tumor samples

A total of 285 formalin-fixed paraffin-embedded (FFPE) tumor samples were obtained from several sources (Sheba Medical Center, Tel-Hashomer, Israel; Soroka University Medical Center, Beer-Sheva, Israel; Beilinson Hospital, Rabin Medical Center, Petah-Tikva, Israel; ABS Inc., Wilmington, DE; Tel Aviv Sourasky Medical Center, Tel Aviv, Israel). The study protocol was approved by the Research Ethics Board of each of the contributing institutes. Each of the FFPE samples was evaluated by a pathologist for histological type, grade and tumor percentage based on hematoxylin-eosin-stained slides, performed on the first and/or last sections of the sample. The tumor content was ≥50% in 92% of the samples. A total of 252 of the samples were profiled by microRNA microarray. Fourteen of these samples and 33 additional samples were profiled by qRT-PCR. Histological classification of the study samples is summarized in Table 1 and listed in detail in Table S1 and Table S2 in Supplementary Information online.

**Table 1:** Summary of samples. Abbreviation: BPH = Benign prostatic hyperplasia.

n	Samples in microarray data—by category	Detail
15	Brain primary tumors	Anaplastic astrocytoma (2), anaplastic oligodendroglioma (1), glioblastoma multiforme (7), low grade astrocytoma (3), oligodendroglioma (2)
187	Other primary tumors	Adipose liposarcoma (4), Bladder (1 transitional cell carcinoma), Breast (3 including 1 infiltrating lobular carcinoma), Cervix (3 adenocarcinoma, 2 squamous cell carcinoma), Colon (4 adenocarcinoma), Endometrium (7 adenocarcinoma), Esophagus (2 adenocarcinoma, 5 squamous cell carcinoma), Esophagus-stomach (7 adenocarcinoma), Gallbladder (3 adenocarcinoma), Kidney (6 renal cell carcinoma), Larynx (4 squamous cell carcinoma), Liver (2 hepatocellular carcinoma), Lung (7 neuroendocrine carcinoid, 1 neuroendocrine large cell, 1 neuroendocrine; mix small cell-large cell, 7 neuroendocrine small cell, 8 non-small cell adenocarcinoma, 3 non-small large cell carcinoma, 8 non-small squamous cell carcinoma, 7 pleura mesothelioma), Lymphocytes (10 Hodgkin's lymphoma), Melanocytes (3 malignant melanoma), Meninges (8 meningioma, 1 atypical meningioma), Mouth (5 squamous cell carcinoma), Nose (5 squamous cell carcinoma), Ovary (7 serous papillary cancer), Pancreas (3 adenocarcinoma, 2 ductal adenocarcinoma, 2 exocrine adenocarcinoma), Prostate (7 samples including 2 BPH samples) Small intestine (7 stromal tumor, 1 adenocarcinoma), Stomach adenocarcinoma (5), Testis seminoma (3), Thymus thymoma (3 type b2, 4 type b3), Thyroid (4 carcinoma, 3 papillary carcinoma, 1 papillary tall cell carcinoma), Tongue (10 squamous cell carcinoma),
50	Metastases in brain	Bladder (1 transitional cell carcinoma), Breast (4 adenocarcinoma, 9 infiltrating ductal carcinoma), Colon (5 adenocarcinoma), Endometrial tumor (1), Kidney (2 clear cell carcinoma, 1 renal cell carcinoma), Lung (10 including 1 carcinoma, 1 neuroendocrine small-cell carcinoma, 6 non-small cell adenocarcinoma, 1 non-small squamous cell carcinoma), Melanocytes (4 melanoma, 2 malignant melanoma), Unknown (3 carcinoma, 5 adenocarcinoma, 1 small cell carcinoma, 2 sarcoma),

n	Additional samples in qRT-PCR validation set	Detail

15	Brain primary tumors	Anaplastic oligodendroglioma (1), astrocytoma (5), glioblastoma multiforme (2), oligodendroglioma (7)
8	Other primary tumors	Bladder (1 transitional cell carcinoma), Kidney (1 renal cell carcinoma), Liver (1 hepatocellular carcinoma), Lung (1 non-small cell adenocarcinoma, 1 pleura mesothelioma), Ovary (1 adenocarcinoma), Pancreas (1 neuroendocrine carcinoma), Thymus thymoma (1 type b2)
10	Metastases in brain	Breast (2 adenocarcinoma), Kidney (3 adenocarcinoma), Lung (1 non-small cell adenocarcinoma, 2 non-small squamous cell carcinoma), Ovary (2 adenocarcinoma)

### RNA extraction

Total RNA was isolated from 7 to 10 10 µm-thick tissue sections per case using the miRdictor™ extraction protocol developed at Rosetta Genomics (Rehovot, Israel). Briefly, the sample was incubated a few times in xylene at 57°C to remove excess paraffin, and then was washed several times with ethanol. Proteins were degraded by incubating the sample in a proteinase K solution at 45°C for a few hours. The RNA was extracted using acid phenol/chloroform and then precipitated using ethanol; DNAses were introduced to digest DNA. Total RNA quantity and quality was measured by Nanodrop™ ND-1000 (NanoDrop Technologies, Wilmington, DE).

Figures S1 and S2 in Supplementary Information online demonstrate the reliability of the RNA extraction protocols.

### miRdicator™ array platform

Custom microRNA microarrays were prepared as described previously (21). Briefly on Slide E coated microarray slides (Schott Nexterion, Mainz, Germany) ∼650 DNA oligonucleotide probes representing microRNAs (Sanger database version 9 and additional microRNAs predicted and validated by Rosetta Genomics) were spotted in triplicate using the BioRobotics MicroGrid II microarrater (Genomic Solutions, Ann Arbor, MI) according to the manufacturer's directions. Fifty-four negative control probes were designed using the sense sequences of different microRNAs. Two types of positive control were included in the experimental design: (i) synthetic small RNAs were spiked into each RNA sample before labeling to verify labeling efficiency; and (ii) probes for abundant small RNAs were spotted on the miRdicator™ array to validate RNA quality.

A total of 3.5 µg of total RNA were labeled by ligation of an RNA-linker, p-rCrU-Cy/dye (Dharmacon, Lafayette, CO; Cy3 or Cy5) to the 3′ end. Slides were incubated with the labeled RNA for 12–16 h at 42°C and then washed twice. Arrays were scanned using Agilent DNA Microarray Scanner Bundle (Agilent Technologies, Santa Clara, CA) at a resolution of 10 µm at 100% power. Array images were analyzed using SpotReader software (Niles Scientific, Portola Valley, CA). Figure S3 demonstrates the reproducibility, sensitivity and specificity of the miRdicator™ microRNA microarray platform.

Microarray spots were combined and signals normalized as described previously (21). Triplicate spots were combined into one signal by taking the logarithmic mean of the reliable spots. All data were log-transformed and the analysis was performed in log-space. A reference data vector for normalization, *R*, was calculated by taking the median expression level for each probe across all samples. For each sample *k* with data vector *S^k^*, a 2nd degree polynomial *F^k^* was found so as to provide the best fit between the sample data and the reference data, such that *R* ≈ *F^k^*(*S^k^*). Remote data points (“outliers”) were not used for fitting the polynomials *F*. For each probe in the sample (element 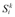
in the vector *S^k^*), the normalized value (in log-space) 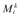
is calculated from the initial value 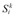
by transforming it with the polynomial function *F^k^*, so that 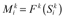. Data is translated back to linear-space by taking the exponent. Henceforth, the expression level or signal of a microRNA refers to the normalized value. Values of normalized expression for each sample for the microRNAs examined in the study are available in Table S1 in Supplementary Information online.

### qRT-PCR

One µg of total RNA was subjected to polyadenylation reaction as described before (25). Briefly, RNA was incubated in the presence of poly (A) polymerase (Takara-2180A), MnCl2 and ATP for 1 h at 37°C. Reverse transcription was performed on the polyadenylated product. An oligo-dT primer harboring a consensus sequence (complementary to the reverse primer) was used for reverse transcription reaction. The primer is first annealed to the poly A–RNA and then subjected to a reverse transcription reaction of SuperScript II RT (Invitrogen). The cDNA was then amplified by real-time PCR reaction, using a miRNA-specific forward primer, TaqMan probe and universal reverse primer. The reactions were incubated for 10 minutes at 95°C, followed by 42 cycles of 95°C for 15 s and 60°C for 1 minute in Applied Biosystems 7500 thermocyclers. Values of cycle to pass threshold (*C_t_*), representing inverse log_2_ expression levels, are listed in Table S2 in Supplementary Information online. Normalizing the C_t_ values (per sample) by the C_t_ of either U6 snRNA(28), the C_t_ of hsa-miR-24, or their average C_t_, shifted at most one sample from each side in the test-set classification predictions.

### Data analysis and statistics

In order to identify microRNA signatures that can be used to differentiate primary brain tumors from brain metastases, we compared the brain primary tumor samples to other primary tumors and to the brain metastases using statistical tests (see Table 2). *P*-values were calculated using a two-sided *t*-test on the log-transformed normalized signal. After adjustment for false detection rate or the more strict Bonferroni correction (multiply each *P*-value by the number of microRNAs tested, ∼1000), most *P*-values remain highly significant (Table 2 lists unadjusted *P*-values; *P*-values are mostly below 1e-8). The *t*-test is designed to identify differences in the distribution mean, but is not an ideal tool to develop classifiers. We used the area under curve (AUC) of the response operating characteristic (ROC) curve to identify microRNA and microRNA combinations that could be used to classify samples accurately (see Figure S5). The receiver operating characteristic curve (ROC curve) plots the sensitivity against the false-positive rate (one minus the specificity) for different cutoff values of a diagnostic metric, and is a measure of classification performance. The area under the ROC curve, or AUC, can be used to assess the diagnostic performance of a metric. A random classifier has AUC = 0.5, and an optimal classifier with perfect sensitivity and specificity of 100% has AUC = 1.

**Table 2:** Comparison between microRNA expression in primary brain tumors and expression in other primary tumors or expression in brain metastases, based on microRNA microarray data. Abbreviation: AUC = area under curve.

Primary brain vs.	Other primary tumors			Brain metastases		
	*P*-value†	fold-change‡	AUC	*P*-value†	fold-change‡	AUC
hsa-miR-124	1.4E-54	97.1	0.9975	5.4E-06	12.6	0.8600
hsa-miR-219-5p	9.7E-43	10.0	0.9679	4.1E-09	6.9	0.8840
*C_0_*†	1.8E-49	293.0	1.0000	9.0E-09	27.7	0.8987
hsa-miR-128	5.4E-27	9.3	0.9929	4.5E-11	4.2	0.9507
hsa-miR-9*	1.4E-64	31.3	1.0000	9.1E-22	18.9	0.9933
hsa-miR-92b	1.8E-26	7.3	0.9993	2.1E-18	5.8	1.0000
*C_1_*†	1.7E-57	205.9	1.0000	3.3E-26	128.7	1.0000

† *P*-values are calculated on log-signal of microRNAs, and on *C_0_* and *C_1_* (methods), which are in log-space. Less that 1000 probes were tested, and even after the more severe Bonferroni correction (multiplying each *P*-value by ∼1000), the *P*-values remain highly significant.

‡ The fold change is calculated by dividing the median signal in brain primary tumors by the median signal in other tissues.

The combined metric *C_0_* was defined as the summed log_2_ expression measured by microarray of hsa-miR-124 and hsa-miR-219-5p: *C_0_* ≡ [log_2_(hsa-miR-124 signal) + log_2_(hsa-miR-219-5p signal)], and had AUC = 1 when used to identify primary brain tumors from other primary tumors, but had AUC = 0.8987 when used to identify brain primary tumors from brain metastases (Figure S5 in Supplementary Information). The combined metric *C_1_* was defined as the summed log_2_ expression measured by microarray of hsa-miR-9* and hsa-miR-92b: *C_1_* ≡ [log_2_(hsa-miR-9* signal) + log_2_(hsa-miR-92b signal)], and had AUC = 1 when used to identify primary brain tumors from other primary tumors or from brain metastases. The calculated values of *C_0_* and *C_1_* for each sample are listed in Table S1 in Supplementary Information online.

The combined metric *C^RT^* was defined as the summed log_2_ expression levels measured by qRT-PCR data (the C_t_ values) of hsa-miR-9 and hsa-miR-92b: *C^RT^* ≡ 100 − [C_t_(hsa-miR-9) + C_t_(hsa-miR-92b signal)], had AUC = 1 in the training set data and one error in the test-set data when used to identify primary brain tumors from other primary tumors or from brain metastases. The combined metric C^RT*^ was defined as the summed qRT-PCR C_t_ values of hsa-miR-9* and hsa-miR-92b: C^RT*^ ≡ 100 − [C_t_(hsa-miR-9*) + C_t_(hsa-miR-92b signal)], had AUC = 1 in the training set data and one error in the test-set data when used to identify primary brain tumors from other primary tumors or from brain metastases. The calculated values of *C^RT^* and C^RT*^ for each sample are listed in Table S2 in Supplementary Information online.

## RESULTS

**Figure 1:**
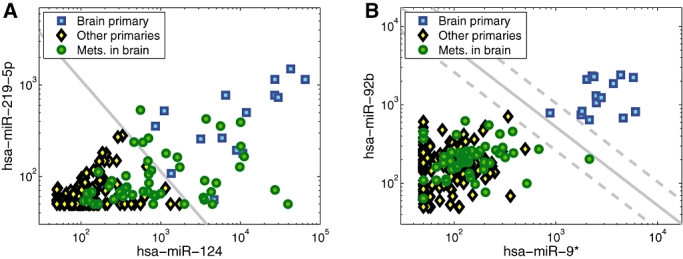
*Identification of metastatic brain tumors using microRNA microarray data*. **A.** Expression levels of hsa-miR-124 and hsa-miR-219-5p in 15 brain primary tumors (blue/cyan squares), 187 primary tumors from other tissues (black/yellow diamonds) and 50 brain metastases originating from various tissues (green circles). Expression levels of hsa-miR-124 and hsa-miR-219-5p are higher in brain primary tumors compared to primary tumors from other tissues. The solid line marks the line where *C_0_* ≡ [log_2_(hsa-miR-124) + log_2_(hsa-miR-219-5p)] = 16.8, and provides perfect separation between brain primary and other primary tumors. The expression levels of hsa-miR-124 and hsa-miR-219-5p in metastatic samples span a wide range on both sides of the separating line. **B.** Expression levels of hsa-miR-9* and hsa-miR-92b in the same samples. Expression levels of these microRNAs are high in brain primary tumors but are low in all other samples. The solid line marks the line where *C_1_* ≡ [log_2_(hsa-miR-9*) + log_2_(hsa-miR-92b)] = 19, and provides perfect separation between brain primary tumors and other samples, including other primary tumors and metastases to the brain. The dashed lines mark a confidence range of factor 2 above or below, *C_1_* = 20 (upper line) and *C_1_* = 18 (lower line). Only two of the samples (<1%) fall within the low-confidence range.

We profiled microRNA expression levels on a microarray platform in 252 tumor samples including 15 brain primary tumor samples, 187 non-brain primary tumors, and 50 brain metastases from various tissue origins (see summary in Table 1 and detail in Table S1 in Supporting Information online). We compared the brain primary tumor samples to the other primary tumor samples and to samples of brain-located metastases (Table 2 and methods). Hsa-miR-124, which is highly specific to the nervous system (14), displayed the greatest disparity in expression when comparing brain primary tumors to other primary tumors, with a fold-change of ∼100 (*P*-value = 5.1e-57, AUC = 0.9976, see Table 2 and Methods). A combination of hsa-miR-124 and hsa-miR-219-5p (*C_0_*, see methods) could be used to distinguish brain primary tumors from non-brain primary tumors with 100% accuracy (Figure 1A, see Table S1 in Supporting Information for values). Other brain-specific microRNAs such as hsa-miR-128 also showed very strong differential expression between brain primary tumors and other primary tumors (*P*-value < 4e-28, AUC = 0.9932, see Figure S4A in Supporting Information online). In extracting and profiling microRNA from bulk tissue samples, the measured RNA sample contains RNA from the tumor cells, but also RNA from the surrounding tissue. In our study, more than 90% of the samples had a tumor content of at least 50%; nevertheless, a fair amount of non-tumor cells are present in the specimens. These microRNAs, which are highly expressed in normal brain (14), were also found at high levels in RNA extracted from brain metastases (Figure 1A). This latter effect, ostensibly caused by contamination from the adjacent normal brain tissue, limits the utility of these microRNAs to serve as biomarkers for differentiating between brain primary tumors and brain-located metastases (AUC of 0.85∼0.95, see Table 2 and Figure S5).

We observed that in addition to the aforementioned microRNAs (hsa-miR-124, hsa-miR-219-5p and hsa-miR-128), hsa-miR-9* and hsa-miR-92b are expressed specifically in brain tumors and not expressed in other tumor types (Figure 1B and Table 2: AUC > 0.99). Importantly, these two microRNAs also differentiate accurately between brain primary tumors and metastatic tumors located in the brain (*P*-value < 3e-18, AUC > 0.99 for each). Indeed, using a combination of hsa-miR-9* and hsa-miR-92b expression (*C_1_*, see methods) it is possible to distinguish brain primary tumor samples from all other samples with 100% accuracy in the microarray data (Figure 1B and Table 2). A simple decision rule, “classify as primary brain tumor if *C_1_* > 19, classify as other if *C_1_* ≤ 19,” identifies correctly all samples. A more conservative classifier can be defined by allowing a margin for uncertainty of factor 2 above or below the threshold (equivalent to one cycle in qRT-PCR measurements). The classification rule “classify as brain primary if *C_1_* > 20, classify as other if *C_1_* < 18, leave unidentified if 18 ≤ *C_1_*≤ 20” leaves only two samples out of 252 (<1%) as unclassified (Figure 1B, see Table S1), and classifies correctly all other samples.

**Figure 2:**
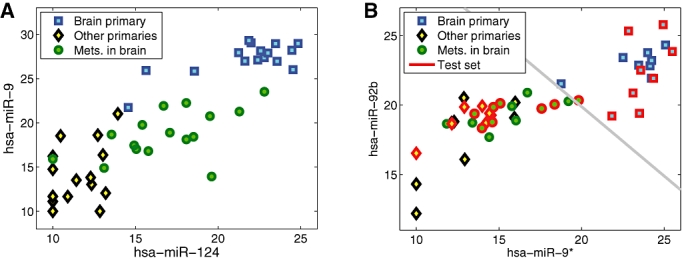
*Identification of metastatic brain tumors using microRNA qRT-PCR data*. **A.** Expression levels (50−C_t_) of hsa-miR-124 and hsa-miR-9 in 16 brain primary tumors (blue/cyan squares), 15 primary tumors from other tissues (black/yellow diamonds) and 16 brain metastases originating from various tissues (green circles). Expression levels of hsa-miR-124 and hsa-miR-9 are higher in brain primary tumors compared to primary tumors from other tissues. The expression levels of hsa-miR-124 in metastatic samples span a wide range and are more similar to brain primary tumors; the expression levels of hsa-miR-9 in metastatic samples are more similar to the non-brain primary tumors. **B.** Expression levels (50−C_t_) of hsa-miR-9* and hsa-miR-92b in the same samples. Expression levels of these microRNAs are high in brain primary tumors and lower in all other samples. The solid line marks C^RT*^ ≡ 100−[C_t_(hsa-miR-9*) + C_t_(hsa-miR-92b)] = 39.9, a threshold that was fit to the training set half of the data. The test-set samples (symbols with red outline) were accurately classified by this threshold, with one outlier. Data points with C_t_ larger than 40 are shown with C_t_ = 40, at (50−C_t_) = 10.

To validate these findings, we profiled 14 of these samples and 33 additional samples by qRT-PCR (Table S2), for four potential biomarkers: hsa-miR-124, hsa-miR-9, hsa-miR-9* and hsa-miR-92b (Table 3), and two controls: hsa-miR-24, which was found to be relatively constantly expressed in the microarray data, and snRNA U6. These microRNAs showed the same pattern as observed in the microarray data (Table 3). Hsa-miR-124 showed strong expression in the brain primary tumors, weak expression in other primary tumors and intermediate expression in the metastases (Figure 2A). Thus, hsa-miR-124 was not a good candidate for identifying metastatic tumors to the brain. On the other hand, hsa-miR-9, hsa-miR-9* and hsa-miR-92b showed specific strong expression in primary brain tumors with lower expression in other tumors and in metastases to the brain (Figure 2), with significant differences and strong separability between brain primary tumors and brain metastases (Table 3).

**Table 3:** Comparison between microRNA expression in primary brain tumors and expression in other primary tumors or expression in brain metastases, based on microRNA qRT-PCR data. Abbreviation: AUC = area under curve.

Primary brain vs.	Other primary tumors			Brain metastases		
	*P*-value†	fold-change‡	AUC	*P*-value†	fold-change‡	AUC
hsa-miR-124	4.7E-9	2144	1.0000	1.4E-4	48	0.8633
hsa-miR-9	2.3E-11	17 648	1.0000	2.0E-11	543	0.9833
hsa-miR-9*	1.5E-11	1887	1.0000	1.4E-12	415	0.9922
hsa-miR-92b	1.7E-6	16	0.9542	7.2E-7	8	0.9219
*C^RT^*	1.1E-10	2.9E+5	1.0000	7.7E-12	9993	0.9961
C^RT^*	2.8E-10	11 868	1.0000	6.4E-12	2428	1.0000

† *P*-values are calculated on measured C_t_ values and on C^RT^ and C^RT^* (methods), which are in log-space. Here only the listed four potential biomarkers and two combinations were tested, and no correction for multiple hypothesis testing is needed.

‡ The fold change is calculated by converting the data to linear space (by taking the exponent base 2) and dividing the median signal in brain primary tumors by the median signal in other tissues.

We defined combinations of hsa-miR-92b with either hsa-miR-9 (*C^RT^*) or with hsa-miR-9* (C^RT*^) by summing their qRT-PCR C_t_ values (see Methods). We selected a threshold for classification for each combination using half of the samples as a training set. We then tested the classification accuracy on the second half of the data set that was used as a test-set. The classifications on the test-set were near perfect with one outlier of 23 samples, reaching 100% accuracy in identifying non-brain primary tumors from brain primary tumors, and 88% sensitivity with 100% specificity in identifying metastatic brain tumors from brain primary tumors, for both C^RT*^ (Figure 2B) and *C^RT^* (Table S2). Indeed, these combinations show significant differences in expression that can be used to classify primary from metastatic brain tumors (Table 3).

According to these data, we propose that hsa-miR-9/9* and hsa-miR-92b, and their combination, represent new biomarkers that can be used to classify brain malignancies—primary vs. secondary.

Hsa-miR-92b and hsa-miR-9/9* were reported previously to be expressed in brain tumors and in cell lines derived from brain tumors (14) and were documented to be expressed specifically in the developing nervous system (12, 13, 32, 34). The gene encoding hsa-miR-9/9* appears in the human genome in three places, in chromosomes 1, 5 and 14, each an identical copy. Hsa-miR-92b is found on chromosome 1 and differs by only one nucleotide in its first 20 from hsa-miR-92a, a member of the oncogenic miR-17-92 cluster (9). However, the expression pattern of hsa-miR-92a did not correlate with that of and hsa-miR-92b (Figure S4C) and was not useful in identification between primary and metastatic brain malignancies.

## DISCUSSION

When faced with a neoplastic brain specimen considered most likely to be a metastasis, a surgical pathologist must always rule out the possibility that it represents a malignant glioma. As a rule, certain morphological features aid in the distinction between secondary and primary neoplasms of the CNS (1). Metastases retain a cohesive quality as they enlarge, and thus remain demarcated from the host tissue, while astrocytic and oligodendroglial tumors exhibit an infiltrative growth pattern, intermingling with the normal cells of the brain tissue. Additionally, in contrast to neoplastic glial cells, neoplastic cells of metastatic carcinomas reveal their epithelial nature by their round or polygonal shape, distinct cellular borders and lack of processes. Furthermore, the amount of fibrous tissue in metastatic lesions is typically greater than in most gliomas, and also, the pattern of necrosis seems to be different in primary tumors from that seen in metastases. However, poorly differentiated metastatic carcinoma may be difficult to distinguish histologically from high-grade astrocytic malignant neoplasms, particularly on small open or stereotactic biopsy specimens.

Immunohistochemical studies are often employed to help in diagnosing brain specimens. One study reported a sensitivity of 100% at a specificity of 86% for identifying primary glioblastoma multiforme (GBM) from metastatic carcinoma using immunohistochemical staining for glial fibrillary acidic protein (GFAP), but a significant number of the metastatic cancers are also stained (17). A combination of immunostains, including GFAP and cytokeratin CAM5.2, has been suggested as useful in differentiating poorly differentiated metastatic carcinoma from GBM (17). However, GBM may variably stain with cytokeratin immunomarkers, usually expressed in epithelial tumors. Co-expression of GFAP and cytokeratin is frequently found, especially in cases of undifferentiated and high grade gliomas (8, 16). Therefore, cytokeratin positivity does not rule out the diagnosis of a glial neoplasm (8). Suggested workup for the differential diagnosis between primary brain neoplasms and poorly differentiated metastatic carcinoma based on immunohistochemistry, therefore, requires a combination of multiple factors, and their accuracy in different scenarios has not yet been established. Regardless of these morphological and immunohistological tools, some neoplastic brain specimens still cannot be definitively classified (19). For instance, anaplastic oligodendroglioma can be macroscopically discrete and composed of compactly arranged polygonal cells such that it simulates a metastatic tumor (26). Despite the progress recently made for molecular evaluation of brain tumors (20, 24, 29), not much data was achieved for the molecular differential diagnosis between primary and metastatic tumors in the brain.

In this study, we present a new diagnostic tool to aid in the differentiation between primary and secondary neoplasms of the CNS: the combined expression of two specific microRNAs, which serves as a novel “brain primary tumor” biomarker.

To find microRNA biomarkers that classify brain tumors, we compared microRNA expression profiles of brain primary tumors, non-brain primary tumors and brain metastases. Initial analyses identified microRNAs, such as hsa-miR-124, that are significantly over-expressed in brain primary tumors compared to non-brain primary tumors, and thus appear to distinguish brain tumors from non-brain tumors (Figure 1A). However, many of these microRNAs are highly expressed in normal brain tissue. Inevitably, during biopsy procedures, significant amounts of normal tissue are excised along with cancerous tissue. In our samples, the tumor content was greater than or equal to 50% in more than 90% of the samples. Nevertheless, as in most studies of expression profiling on tissue samples, a fair amount of non-tumor cells are present in the specimens. Thus, brain-specific microRNAs, such as hsa-miR-124, can be found variously in tissue specimens containing brain metastases, and their expression levels measured in bulk tissue samples do not allow discrimination between brain primary tumors and metastases located in the brain.

Further analyses, which included a substantial set of 50 brain metastasis samples, delineated a subset of microRNAs that are expressed specifically in brain primary tumors, but importantly, neither in non-brain primary tumors nor in brain-located metastases. Specifically, we found that hsa-miR-92b and hsa-miR-9/9* are very significantly and strongly over-expressed in samples of primary brain tumors, but not in samples of metastatic tumors to the brain. These bulk samples contain surrounding tissue, and it cannot be ruled out at this stage that a fraction of the over-expressed microRNAs may be derived from non-neoplastic glial cells in the surrounding tissue, in which this expression is specific to reactive cells surrounding primary tumors only. Regardless of its biological origin, the specific over-expression of these microRNAs in samples from brain primary tumors provides important diagnostic information. The combined expression levels of hsa-miR-92b and hsa-miR-9/9* allow discrimination between brain primary tumors and metastases located in the brain with very high accuracy, and thus represent a potential biomarker for the identification of brain primary tumors.

Notably, hsa-miR-92b and hsa-miR-9/9* were reported previously to be over-expressed in neuronal-specific stem cells and to exhibit dynamic expression patterns in the developing brain (12, 13, 32, 34). Thus, it appears that elevated expression of these microRNAs is a feature common to brain stem cells and brain tumor cells. Whether cancerous cells originate from abnormal stem cells or represent de-differentiated somatic cells is a hotly debated issue (4, 31). In any case, our data reinforce the enigmatic association between pluripotency and tumorigenesis. Further research should elucidate the function of these microRNAs in the brain and their roles in brain cancer.

## DISCLOSURE

All authors affiliated with Rosetta Genomics are full-time employees of Rosetta Genomics Ltd. and hold equity in the company, the value of which may be influenced by this publication.

## SUPPLEMENTARY MATERIAL

The following supplementary material is available for this article:

**Figure S1. A.** RNA was extracted and profiled (4 μg total RNA) from a mouse liver sample, which was either preserved as fresh frozen (*y*-axis) or formalin-fixed, paraffin-embedded (FFPE) (*x*-axis). Overall correlation of expressions was 0.9. Similar results were obtained for RNA extracted from lung. **B.** Similar results were obtained when comparing microRNA expression extracted from FFPE sample (*x*-axis) to the RNA extracted from formalin-fixed sample (*y*-axis). Overall correlation of expressions was 0.9. Similar results were obtained for RNA extracted from lung. We next examined the difference in the level of microRNA expression in tissues (liver and lung) preserved at different time points, immediate, 1 h and 5 h post-operation. The microRNA fraction in the lung was not affected even when kept non-fixed for 5 h. Delaying fixation of a liver sample by 1 h had no significant change on the microRNA fraction, but the microRNA fraction was decreased approximately twofold when the liver sample was kept non-fixed for 5 h, reflecting the high content of nucleases in liver tissue (data not shown).

**Figure S2.** MicroRNA stability in formalin-fixed, paraffinembedded (FFPE) archival samples of different ages: total RNA was extracted from 13 lung FFPE samples of different ages (ages 2–11 years) and hybridized to the miRdicator™ microarray. Each bar in the graph depicts the overall microRNA expression correlation (Pearson correlation coefficient) between a single sample (age appearing on the *x*-axis), and the newest sample (age 2 years). The high correlation (mostly above 0.9) indicates the highly preserved fraction of microRNA in paraffin blocks even after many years.

**Figure S3. A.** Microarray reproducibility: 3 μg of placenta RNA (Ambion) was repeatedly (seven times) labeled and hybridized to the microarray. For >200 highly expressed microRNAs (sorted from top to bottom by mean expression level), each column depicts the expression in one repeat. For each pair of repeats, the Pearson correlation coefficient was computed, resulting in an overall mean correlation coefficient of 0.99. **B.** Microarray sensitivity and dynamic range: four synthetic short RNAs, 22 nt long, were spiked in different amounts into 3 μg of placenta total RNA, labeled and hybridized to the array. Each line corresponds to one of the spike-ins. The lowest sensitivity was found to be 0.1 fmole with a linear dynamic range of about three orders of magnitude. **C.** Microarray specificity: synthetic RNAs of hsalet- 7a, c and d were spiked into non-relevant background material (high molecular weight RNA extracted from HeLa cell line, which shows no hybridization background when hybridized to the microarray, data not shown) and hybridized to the miRdicator™ microarray. The table depicts the probe signals of the let-7 family in response to the spiked-in synthetic RNAs (columns), normalized by the signal of the probe corresponding to the synthetic RNA. Specificity of about 10-fold in the signal level between let-7a and let-7c was demonstrated, representing the level of specificity for a single nucleotide mismatch. **D.** Tissue specificity: 3–5 μg of total RNA extracted from liver and testis formalinfixed, paraffin-embedded samples were labeled and hybridized to the miRdicator™ microarray, identifying tissue specific microRNA expression profiles such as hsa-miR-122a in the liver and hsa-miR-372 and hsa-miR-373 in the testis tissue.

**Figure S4.** *Expression levels of microRNA (microarray data) in 15 primary brain tumors (blue/cyan squares), 187 primary tumors from other tissues (black/yellow diamonds) and 50 brain metastases from various tissue origins (green circles)*. **A.** HsamiR-128 has high expression levels in brain primary tumors, low in other primary tumors and intermediate in brain metastasis samples (see Table 2). In contrast to hsa-miR-128, hsa-miR-92b is specifically expressed in brain primary tumors, and is lower in primary tumors from other tissues and in their metastases to the brain. **B.** Hsa-miR-124 has high expression levels in brain primary tumors, low in other primary tumors, and its expression levels in RNA extracted from brain metastasis samples spans a wide range. In contrast to hsa-miR-124, hsa-miR-9* is specifically expressed in brain primary tumors, and is lower in primary tumors from other tissues and in their brain metastases. **C.** Expression of hsa-miR-92a did not correlate with expression of hsa-miR-92b and was not useful for identification between primary and metastatic brain malignancies.

**Figure S5.** *The receiver operating characteristic curve (ROC curve) plots the sensitivity against the false-positive rate (one minus the specificity) for different cutoff values of a diagnostic metric, and is a measure of classification performance. The area under the ROC curve [area under curve(AUC)] can be used to asses the diagnostic performance of metric. A random classifier has AUC = 0.5, and an optimal classifier with perfect sensitivity and specificity of 100% has AUC = 1.* **A.** The sorted values of *C_0_* for 15 samples of brain primary tumors (blue squares) and 187 samples of non-brain primary tumors (red circles). The dashed line shows *C_0_* = 16.8, which perfectly separates these two sets of samples (sensitivity = specificity = 100%). **B.** The ROC curve for *C_0_* in these samples, which has AUC = 1. **C.** The sorted values of *C_0_* for 15 samples of brain primary tumors (blue squares) and 50 samples of brain metastases (red circles). The dashed line shows *C_0_* = 16.8, which misclassifies 20 of the 50 metastasis samples (sensitivity = 60%, specificity = 100%). *D.* The ROC curve for *C_0_* in these samples, which has AUC = 0.8987.

**Table S1.** Details for the 252 samples measured by microarray, expression levels of microRNAs used in the study, and combined metrics C_0_ and C_1_.

**Table S2.** Details for the 47 samples measured by qRT-PCR, expression levels of microRNAs used in the study, and combined metrics C^RT^ and C^RT*^.

This material is available as part of the online article from: http://www.blackwellsynergy.com
