# Supplementary material for: MiR-92b and miR-9/9* Are Specifically Expressed in Brain Primary Tumors and Can Be Used to Differentiate Primary from Metastatic Brain Tumors
Source: Brain Pathol. 2009 Jul;19(3):375–83. doi: 10.1111/j.1750-3639.2008.00184.x (PMC2728890; doi:10.1111/j.1750-3639.2008.00184.x)
Supplement: Supplementary file 1 [file bpa0019-0375-SD1.doc]

**MiR-92b and miR-9/9* are specifically expressed in brain primary tumors and can be used to differentiate primary from metastatic brain tumors**

Nass, Rosenwald et al. 2008

**Supplementary Information**

**Supplementary Figures**

**Figure S1**

**a**

**b**

A) RNA was extracted and profiled (4 μg total RNA) from a mouse liver sample which was either preserved as fresh-frozen (y-axis) or FFPE (x-axis). Overall correlation of expressions was 0.9. Similar results were obtained for RNA extracted from lung.

B) Similar results were obtained when comparing microRNA expression extracted from FFPE sample (x-axis) to the RNA extracted from formalin-fixed sample (y-axis). Overall correlation of expressions was 0.9. Similar results were obtained for RNA extracted from lung. We next examined the difference in the level of microRNA expression in tissues (liver & lung) preserved at different time points, immediate, 1h & 5h post operation. The microRNA fraction in the lung was not affected even when kept non-fixed for 5 hours. Delaying fixation of a liver sample by 1 hour had no significant change on the microRNA fraction, but the microRNA fraction was decreased approximately 2-fold when the liver sample was kept non-fixed for 5 hours, reflecting the high content of nucleases in liver tissue (data not shown).

**Figure S2**

MicroRNA stability in FFPE of different ages: total RNA was extracted from 13 lung FFPE samples of different ages (ages 2-11 years) and hybridized to the miRdicator^TM^ microarray. Each bar in the graph depicts the overall microRNA expression correlation (Pearson correlation coefficient) between a single sample (age appearing on the x-axis), and the newest sample (age 2 years). The high correlation (mostly above 0.9) indicates the highly preserved fraction of microRNA in paraffin blocks even after many years.

**Figure S3**

**A B**

**C D**

A) Microarray reproducibility: 3 μg of placenta RNA (Ambion) was repeatedly (seven times) labeled and hybridized to the microarray. For >200 highly expressed microRNAs (sorted from top to bottom by mean expression level), each column depicts the expression in one repeat. For each pair of repeats the Pearson correlation coefficient was computed resulting in an overall mean correlation coefficient of 0.99.

B) Microarray sensitivity & dynamic range: four synthetic short RNAs, 22 nt long, were spiked in different amounts into 3 μg of placenta total RNA, labeled and hybridized to the array. Each line corresponds to one of the spike-ins. The lowest sensitivity was found to be 0.1 fmole with a linear dynamic range of about three orders of magnitude.

C) Microarray specificity: synthetic RNAs of hsa-let-7a, c & d were spiked into non- relevant background material (high molecular weight RNA extracted from HeLa cell line, which shows no hybridization background when hybridized to the microarray, data not shown) and hybridized to the miRdicator^TM^ microarray. The table depicts the probe signals of the let-7 family in response to the spiked-in synthetic RNAs (columns), normalized by the signal of the probe corresponding to the synthetic RNA. Specificity of about 10-fold in the signal level between let-7a and let-7c was demonstrated, representing the level of specificity for a single nucleotide mismatch.

D) Tissue specificity: 3-5 μg of total RNA extracted from liver and testis FFPE samples were labeled and hybridized to the miRdicator^TM^ microarray, identifying tissue specific microRNA expression profiles such as hsa-miR-122a in the liver and hsa-miR-372 and hsa-miR-373 in the testis tissue.

**Figure S4**

Expression levels of microRNA (microarray data) in 15 primary brain tumors (blue/cyan squares), 187 primary tumors from other tissues (black/yellow diamonds), and 50 brain metastases from various tissue origins (green circles).

A) Hsa-miR-128 has high expression levels in brain primary tumors, low in other primary tumors, and intermediate in brain metastasis samples (see Table 2). In contrast to hsa-miR-128, hsa-miR-92b is specifically expressed in brain primary tumors, and is lower in primary tumors from other tissues and in their metastases to the brain.

B) Hsa-miR-124 has high expression levels in brain primary tumors, low in other primary tumors, and its expression levels in RNA extracted from brain metastasis samples spans a wide range. In contrast to hsa-miR-124, hsa-miR-9* is specifically expressed in brain primary tumors, and is lower in primary tumors from other tissues and in their brain metastases.

C) Expression of hsa-miR-92a did not correlate with expression of hsa-miR-92b and was not useful for identification between primary and metastatic brain malignancies.

**Figure S5**

The receiver operating characteristic curve (ROC curve) plots the sensitivity against the false-positive rate (one minus the specificity) for different cutoff values of a diagnostic metric, and is a measure of classification performance. The area under the ROC curve (AUC) can be used to asses the diagnostic performance of metric. A random classifier has AUC=0.5, and an optimal classifier with perfect sensitivity and specificity of 100% has AUC=1.

A) The sorted values of *C_0_* for 15 samples of brain primary tumors (blue squares) and 187 samples of non-brain primary tumors (red circles). The dashed line shows *C_0_*=16.8, which perfectly separates these two sets of samples (sensitivity=specificity=100%).

B) The ROC curve for *C_0_* in these samples, which has AUC=1.

C) The sorted values of *C_0_* for 15 samples of brain primary tumors (blue squares) and 50 samples of brain metastases (red circles). The dashed line shows *C_0_*=16.8, which misclassifies 20 of the 50 metastasis samples (sensitivity=60%, specificity=100%).

D) The ROC curve for *C_0_* in these samples, which has AUC=0.8987.
